# Comparative View of Mortality from Child-Birth in London and Dublin

**Published:** 1816-09

**Authors:** 


### *For the London Medical and Physical Journal.*

*Comparative View of Mortality from Child-birth in London and Dublin;* by Medicus U.

In the twenty-fifth volume of this Journal, a paper was inserted (page 214), showing the number of deaths in child-bed, taken from the London Bills of Mortality, according to averages calculated upon periods of ten years each. There has lately been published in the Edinburgh Medical and Surgical Journal, No. XLVII. "An Abstract of the Registry kept at the Lying-in Hospital, in Dublin, from the 8th of Dec. 1757 (the day it was first opened) to Dec. 31, 1814;" from which it appears that 78,001 women have been delivered—that the number of children amounted to 79,503, of which 41,523 were boys, and 37,982 girls, which is in the proportion of about 10 males to 9 females—that the number of plural births were 1,372, or 1 in 57, of which 1,350 were twins, 21 triplets, and 1 quadruplet; the proportion of more than two at a birth being 1 in 3,545. The still-born children amounted to 4,329, which is about 1 in 18: the children dying in the Hospital after birth (I presume, in the first month) were 4,810, about 1 in 16. The deaths of women in child-bed were 835 (not 838 as misprinted), which is in the proportion of 1 in 93.

In order to compare the deaths in child-bed at this Hospital with what occurred during the same periods at London, I have been induced to take averages of ten years each, as has been before done in your Journal; and, if you think proper to publish the same, my calculations are much at your service.

| *At the Dublin Lying-in Hospital.* | | *At London.* | |
|---|---|---|---|
| Three years and a few days to Dec. 31, 1760 | 1 in 82 | Ten years to Dec. 31, 1760 | 1 in 81 |
| 10 ys. to Dec. 31, 1770 | 1 in 67 | 1770 | 1 in 72 |
| 1780 | 1 in 94 | 1780 | 1 in 92 |
| 1790 | 1 in 103 | 1790 | 1 in 107 |
| 1800 | 1 in 117 | 1800 | 1 in 113 |
| 1810 | 1 in 102 | 1810 | 1 in 106 |
| 4 yrs. to Dec. 31, 1814 | 1 in 66 | Four years 1814 | 1 in 110 |

To what is the enormous increase of mortality in child-bed at the Dublin Hospital, during the last four years, to be attributed?

*August*, 1816.

